# What We Know, Are Still Getting Wrong, and Have Yet to Learn about the Relationships among the SAT, Intelligence and Achievement

**DOI:** 10.3390/jintelligence7040026

**Published:** 2019-12-02

**Authors:** Meredith C. Frey

**Affiliations:** Department of Psychology, Otterbein University, 1 S. Grove St., Westerville, OH 43081, USA; MFrey@Otterbein.edu

**Keywords:** intelligence, SAT, academic achievement

## Abstract

Fifteen years ago, Frey and Detterman established that the SAT (and later, with Koenig, the ACT) was substantially correlated with measures of general cognitive ability and could be used as a proxy measure for intelligence (Frey and Detterman, 2004; Koenig, Frey, and Detterman, 2008). Since that finding, replicated many times and cited extensively in the literature, myths about the SAT, intelligence, and academic achievement continue to spread in popular domains, online, and in some academic administrators. This paper reviews the available evidence about the relationships among the SAT, intelligence, and academic achievement, dispels common myths about the SAT, and points to promising future directions for research in the prediction of academic achievement.

## 1. Introduction

When I was a first-year graduate student, my advisor was contacted by someone with an interesting problem. A person had suffered a head injury, and there was some indication that the individual’s intelligence had been negatively impacted by the injury. However, the only premorbid measure of intellectual functioning that existed for this person was the SAT, and, while many suspected the SAT was a de facto intelligence test, the literature in the area was surprisingly thin. In two studies, we found that SAT scores correlated up to 0.8 with measures of fluid reasoning ability and *g*, and as highly with traditional intelligence test scores as scores on those tests did with each other. Frey and Detterman established that the SAT (and, with Koenig, the ACT) was *g*-loaded, could be used as a proxy measure for intelligence, and could be converted to an IQ scale with a simple equation [1,2]. In addition to the application of estimating premorbid intelligence, we reasoned that researchers would be interested in establishing this relationship, since it could eliminate time-consuming test administration when they were looking for a measure of intelligence in the context of larger studies (e.g., establishing relationships between intelligence and other traits/abilities). In other words, in answer to the question of this special issue, we found that the SAT measures intelligence.

Frey and Detterman [1] has been cited 286 times, according to the Social Sciences Citation Index, and 651 times by Google Scholar as of the writing of this paper. Now, fifteen years after that work, researchers have confirmed the principal finding, yielding correlations between intelligence and the SAT of roughly 0.5 to 0.9, depending upon sample and the way in which intelligence is defined [3,4,5]. Yet, there is a tendency to conflate clarification or incremental improvement with wholesale ignorance about a topic—an application of a perfectionist fallacy—especially when research moves from scholarly literature to public consumption. Perhaps, then, the real question of this special issue should be: aside from intelligence, what does the SAT measure? Barring that change, we must make every effort to ensure that the established findings remain clear.

## 2. What We Know about the SAT

### 2.1. The SAT Measures Intelligence

Although the principal finding of Frey and Detterman has been established for 15 years, it bears repeating: the SAT is a good measure of intelligence [1]. Despite scientific consensus around that statement, some are remarkably resistant to accept the evidence of such an assertion. In the wake of a recent college admissions cheating scandal, Shapiro and Goldstein reported, in a piece for the *New York Times*, “*The SAT and ACT are not aptitude or IQ tests*” [6]. While perhaps this should not be alarming, as the authors are not experts in the field, the publication reached more than one million subscribers in the digital edition (the article also appeared on page A14 in the print edition, reaching hundreds of thousands more). And it is false, not a matter of opinion, but rather directly contradicted by evidence. 

For years, SAT developers and administrators have declined to call the test what it is; this despite the fact that the SAT can trace its roots through the Army Alpha and Beta tests and back to the original Binet test of intelligence [7]. This is not to say that these organizations directly refute Frey and Detterman; rather, they are silent. On the ETS website, the word intelligence does not appear on the pages containing frequently asked questions, the purpose of testing, or the ETS glossary. If one were to look at the relevant College Board materials (and this author did, rather thoroughly), there are no references to intelligence in the test specifications for the redesigned SAT, the validity study of the redesigned SAT, the technical manual, or the SAT understanding scores brochure. 

Further, while writing this paper, I entered the text “does the SAT measure intelligence” into the Google search engine. Of the first 10 entries, the first (an advertisement) was a link to the College Board for scheduling the SAT, four were links to news sites offering mixed opinions, and fully half were links to test prep companies or authors, who all indicated the test is not a measure of intelligence. This is presumably because acknowledging the test as measure of intelligence would decrease consumers’ belief that scores could be vastly improved with adequate coaching (even though there is substantial evidence that coaching does little to change test scores). One test prep book author’s blog was also the “featured snippet”, or the answer highlighted for searchers just below the ad. In the snippet, the author made the claims that “The SAT does not measure how intelligent you are. Experts disagree whether intelligence can be measured at all, in truth” [8]—little wonder, then, that there is such confusion about the test. 

### 2.2. The SAT Predicts College Achievement

Again, an established finding bears repeating: the SAT predicts college achievement, and a combination of SAT scores and high school grades offer the best prediction of student success. In the most recent validity sample of nearly a quarter million students, SAT scores and high school GPA combined offered the best predictor of first year GPA for college students. Including SAT scores in regression analyses yielded a roughly 15% increase in predictive power above using high school grades alone. Additionally, SAT scores improved the prediction of student retention to the second year of college [9]. Yet many are resistant to using standardized test scores in admissions decisions, and, as a result, an increasing number of schools are becoming “test optional”, meaning that applicants are not required to submit SAT or ACT scores to be considered for admission. But, without these scores, admissions officers lose an objective measure of ability and the best option for predicting student success.

### 2.3. The SAT Is Important to Colleges

Colleges, even nonselective ones, need to identify those individuals whose success is most likely, because that guarantees institutions a consistent revenue stream and increases retention rates, seen by some as an important measure of institutional quality. Selective and highly selective colleges further need to identify the most talented students because those students (or, rather, their average SAT scores) are important for the prestige of the university. Indeed, the correlation between average SAT/ACT scores and college ranking in *U.S. News & World Report* is very nearly 0.9 [10,11].

### 2.4. The SAT Is Important to Students

Here, it is worth recalling the reason the SAT was used in admissions decisions in the first place: to allow scholarship candidates to apply for admission to Harvard without attending an elite preparatory school [7]. Without an objective measure of ability, admissions officers are left with assessing not just the performance of the student in secondary education, but also the quality of the opportunities afforded to that student, which vary considerably across the secondary school landscape in the United States. Klugman analyzed data from a nationally representative sample and found that high school resources are an important factor in determining the selectivity of colleges that students apply for, both in terms of programmatic resources (e.g., AP classes) and social resources (e.g., socioeconomic status of other students) [12]. It is possible, then, that relying solely on high school records will exacerbate rather than reduce pre-existing inequalities.

Of further importance, performance on the SAT predicts the probability of maintaining a 2.5 GPA (a proxy for good academic standing) [9]. Universities can be rather costly and admitting students with little chance of success until they either leave of their own accord or are removed for academic underperformance—with no degree to show and potentially large amounts of debt—is hardly the most just solution. 

## 3. What We Get Wrong about the SAT

Nearly a decade ago, Kuncel and Hezlett provided a detailed rebuttal to four misconceptions about the use of cognitive abilities tests, including the SAT, for admissions and hiring decisions: (1) a lack of relationship to non-academic outcomes, (2) predictive bias in the measurements, (3) a problematically strong relationship to socioeconomic status, and (4) a threshold in the measures, beyond which individual differences cease to be important predictors of outcomes [13]. Yet many of these misconceptions remain, especially in opinion pieces, popular books, blogs, and more troublingly, in admissions decisions and in the hearts of academic administrators (see [14] for a review for general audiences).

### 3.1. The SAT Mostly Measures Ability, Not Privilege

SAT scores correlate moderately with socioeconomic status [15], as do other standardized measures of intelligence. Contrary to some opinions, the predictive power of the SAT holds even when researchers control for socioeconomic status, and this pattern is similar across gender and racial/ethnic subgroups [15,16]. Another popular misconception is that one can “buy” a better SAT score through costly test prep. Yet research has consistently demonstrated that it is remarkably difficult to increase an individual’s SAT score, and the commercial test prep industry capitalizes on, at best, modest changes [13,17]. Short of outright cheating on the test, an expensive and complex undertaking that may carry unpleasant legal consequences, high SAT scores are generally difficult to acquire by any means other than high ability. 

That is not to say that the SAT is a perfect measure of intelligence, or only measures intelligence. We know that other variables, such as test anxiety and self-efficacy, seem to exert some influence on SAT scores, though not as much influence as intelligence does. Importantly, though, group differences demonstrated on the SAT may be primarily a product of these noncognitive variables. For example, Hannon demonstrated that gender differences in SAT scores were rendered trivial by the inclusion of test anxiety and performance-avoidance goals [18]. Additional evidence indicates some noncognitive variables—epistemic belief of learning, performance-avoidance goals, and parental education—explain ethnic group differences in scores [19] and variables such as test anxiety may exert greater influence on test scores for different ethnic groups (e.g., [20], in this special issue). Researchers and admissions officers should attend to these influences without discarding the test entirely. 

### 3.2. The SAT Predicts More Than First Year Grades

After first year grades, the SAT continues to predict college achievement. Higher SAT scores are associated with an increase in the number of advanced courses students take within a degree program, even after controlling for things such as AP credit [21]. In addition, Coyle and colleagues have investigated the utility of *g* and non-*g* variances from the SAT in predicting general college achievement, and performance in specific courses, and found that both *g* and non-*g* variances from the SAT predict these college outcomes [22,23].

We also know that the SAT is useful in predicting life outcomes long after college, even in the most restricted samples. For example, researchers have found that SAT scores in intellectually precocious children at age 13 predicted achievements more than two decades later [24,25]. Further, Wai has determined that ability level (as measured by the average SAT score of the university attended) predicts individual differences in wealth, even among the wealthiest individuals [26].

### 3.3. Why the Resistance to SAT, Intelligence, and Achievement Relationships?

None of the findings reported in this paper are particularly new, yet all seem to be consistently misunderstood or disregarded in public perceptions of the SAT. Recent cheating scandals further perpetuate the notion that high SAT scores are the result of influence, money, or fraud rather than high ability. Although it is unknown exactly why public opinion has coalesced the way it has around the SAT, some findings regarding cross-cultural beliefs about intelligence may shed light on the particular reactivity Americans have to the SAT. Research by Swami and colleagues compared levels of agreement about three domains of findings of intelligence research—(1) Stability, validity, and reliability of intelligence tests, (2) Practical importance of intelligence, and (3) Source and stability of within-group differences—in three countries: the U.S., Britain, and Malaysia. The authors determined that the British and American samples were more skeptical about findings related to the first two domains; that is, they were less likely to believe that intelligence tests were good measures of intelligence and that intelligence has practical importance [27]. If we extend this finding to the SAT as a measure of cognitive ability, the public perception becomes *intelligence isn’t measured by the SAT and SAT scores don’t matter.*

## 4. What Researchers Are Still Learning about the SAT

Hunt and Jaeggi thoughtfully summarized several issues in intelligence research in the inaugural issue of this journal, and those points also apply to the study of the SAT [28]. One problem the authors noted was the distinction between intelligence as “effective cognitive performance” (what intelligent people do) and intelligence as “relative standing on a set of cognitive traits” (or individual differences in intelligence test scores). Certainly, the SAT as a measure of intelligence is primarily concerned with the latter. However, other cognitive traits not measured by the SAT surely influence the effective cognitive performance necessary to be successful in academic settings, leaving a wide swath of intelligent behavior that should be investigated in relationship to academic achievement and other measures of success.

Further, we are still learning about the noncognitive variables that can improve upon the prediction of academic achievement. Research consistently demonstrates that conscientiousness, study habits, and attitudes are important for the prediction of academic achievement [13]; grit also seems to be useful as an incremental predictor in highly competitive environments [29], though more recent meta-analytic evidence shows that grit predicts academic achievement only modestly, and not incrementally over the established traits of cognitive ability and conscientiousness [30]. Finding ways to measure noncognitive traits objectively, avoiding any self-serving bias on the part of applicants, could provide incremental predictive value in admissions decisions.

We know that academic achievement is heritable, and we know that intelligence contributes to that heritability, largely for genetic reasons. Researchers have discovered that some noncognitive factors contributing to the heritability of academic achievement in children are largely genetic, as well [31]. Further, Ayorech, Plomin, and Von Stumm recently demonstrated the importance of gene-environment correlations in predicting educational trajectories in the UK [32]. Taken together, these findings suggest that future research will continue to examine the genetic and environmental influences on cognitive and non-cognitive antecedents to academic success.

Finally, we do not yet know how schools will use SAT’s new Landscape—the replacement for the Environmental Context Dashboard “adversity score”—in supplementing SAT scores in the admissions decisions. After a fair amount of skepticism about the application, utility, and transparency of the adversity score, Landscape was designed to give admissions officers information about applicants’ neighborhoods and schools without giving each applicant a score [33]. Aside from changes to the test and score reports, advances in analysis, rigorous research on instructional practices, and developments in neuroscience will doubtless shape future investigations in predicting academic achievement. Yet, just because there is more work to do in the field does not mean that it is necessary to reject what is known: the SAT measures intelligence and is valuable in predicting college achievement and other important outcomes.

## 5. Conclusions

If we fail to acknowledge what the SAT measures, we give rise to the claims that it measures privilege or test-taking ability or preparation, instead of the important, complex thinking required to do well in college. If we are willing to accept that the SAT measures intelligence relatively well, and that intelligence is useful in college, then we are able to continue to use the assessment as part of an admissions process that identifies individuals who have a good chance of college success, even if they come from underperforming high schools with comparatively weak curricula. As an added benefit, we may begin to rein in the claims of test preparation companies who have made considerable profits from the widely held but erroneous belief that large increases in scores are likely with costly instruction. Finally, when we understand that the SAT is a reasonable measure of intelligence, we can use SAT scores as a proxy measure for time-consuming and sometimes unavailable traditional intelligence assessments, as dozens of researchers have been doing since 2004.
